# Supplementary material for: Parental dietary vitamin B12 causes intergenerational growth acceleration and protects offspring from pathogenic microsporidia and bacteria
Source: iScience. 2024 Jun 6;27(7):110206. doi: 10.1016/j.isci.2024.110206 (PMC11237918; doi:10.1016/j.isci.2024.110206)
Supplement: Document S1. Figures S1 and S2 [file mmc1.pdf]

iScience, Volume *27*

Supplemental information

Parental dietary vitamin B12 causes intergenerational growth acceleration and protects offspring from pathogenic microsporidia and bacteria

Alexandra R. Willis, Winnie Zhao, Ronesh Sukhdeo, Nicholas O. Burton, and Aaron W. Reinke

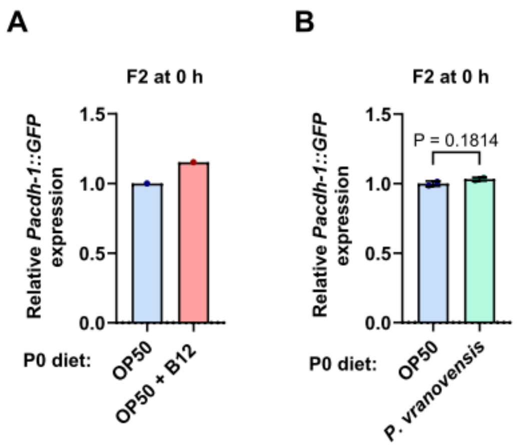

Figure S1. Expression of *acdh-1* is recovered in F2 offspring (related to Figure 1)

(A and B) F1 offspring were all grown on OP50. Levels of *Pachd-1*::*GFP* fluorescence in F2 offspring of worms grown on vitamin B12 (A) or *P. vranovensis* (B), normalized to the offspring of OP50-fed worms at the same timepoint. Data is from n = 1 (A) or n = 2 (B) independent replicates of N = 15 worms quantified per condition per replicate. Horizontal bars represent mean ± SD. Dots represent the mean value of the worms quantified for each replicate. The p-value was determined by unpaired two-tailed Student’s t test.

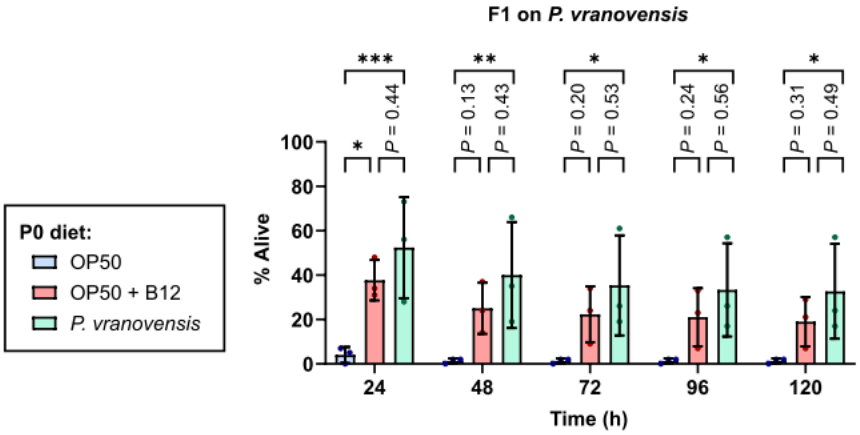

Figure S2. Offspring resistance to *P. vranovensis* remains throughout adulthood (related to Figure 3)

Worms were fed OP50, OP50 supplemented with vitamin B12, or *P. vranovensis*. Offspring embryos were plated on lawns of *P. Vranovensis*. Percentage of initial worms plated surviving after 24 h to 120 h was quantified. Data is from n = 3 independent replicates of N = 77 to 106 worms per condition per replicate. Horizontal bars represent mean ± SD. Dots represent the value for each replicate. The p-values were determined by two-way ANOVA with Tukey's post hoc test. Significance defined as * $p < 0.05$, ** $p < 0.01$, *** $p < 0.001$.
